# Revisiting fetal dose during radiation therapy: evaluating treatment techniques and a custom shield

**DOI:** 10.1120/jacmp.v17i5.6135

**Published:** 2016-09-08

**Authors:** Amir M. Owrangi, Donald A. Roberts, Elizabeth L. Covington, James A. Hayman, Kathryn M. Masi, Choonik Lee, Jean M. Moran, Joann I. Prisciandaro

**Affiliations:** ^1^ Department of Radiation Oncology University of Michigan Ann Arbor MI USA

**Keywords:** peripheral dose, out‐of‐field dose, fetal shield, fetus

## Abstract

To create a comprehensive dataset of peripheral dose (PD) measurements from a new generation of linear accelerators with and without the presence of a newly designed fetal shield, PD measurements were performed to evaluate the effects of depth, field size, distance from the field edge, collimator angle, and beam modifiers for common treatment protocols and modalities. A custom fetal lead shield was designed and made for our department that allows external beam treatments from multiple angles while minimizing the need to adjust the shield during patient treatments. PD measurements were acquired for a comprehensive series of static fields on a stack of Solid Water. Additionally, PDs from various clinically relevant treatment scenarios for pregnant patients were measured using an anthropomorphic phantom that was abutted to a stack of Solid Water. As expected, the PD decreased as the distance from the field edge increased and the field size decreased. On average, a PD reduction was observed when a 90° collimator rotation was applied and/or when the tertiary MLCs and jaws defined the field aperture. However, the effect of the collimator rotation (90° versus 0°) in PD reduction was not found to be clinically significant when the tertiary MLCs were used to define the field aperture. In the presence of both the MLCs and the fetal shield, the PD was reduced by 58% at a distance of 10 cm from the field edge. The newly designed fetal shield may effectively reduce fetal dose and is relatively easy to setup. Due to its design, we are able to use a broad range of treatment techniques and beam angles. We believe the acquired comprehensive PD dataset collected with and without the fetal shield will be useful for treatment teams to estimate fetal dose and help guide decisions on treatment techniques without the need to perform pretreatment phantom measurements.

PACS number(s): 87.53.Bn, 87.55.D‐, 87.55.N

## I. INTRODUCTION

Radiation therapy may be considered for the treatment of malignancies in pregnant patients in situations where treatment cannot be postponed until the postpartum period. Considering the radiosensitive nature of the fetus, special care is required to reduce the fetal dose. The report from AAPM Task Group (TG) 36^(1)^ recommends that the equivalent absorbed dose received by a fetus should be kept below 5 cGy to minimize the risk of adverse biological effects. Depending on the gestational stage and the dose absorbed by the fetus, radiation poses a risk of lethality, malformation, growth retardation, mental retardation, carcinogenesis, genetic abnormalities, and behavioral disorders.^1^, ^2^, ^3^, ^4^ However, these risks can be minimized with careful planning and precautions to ensure the dose to the fetus is as low as reasonably achievable.

The goal of radiotherapy is to deliver a high dose to the defined treatment target in an attempt to control the growth of the tumor while also minimizing dose to normal structures. To achieve the optimum treatment plan that balances the risks and benefits of radiotherapy, deliberate treatment strategies should be used in an attempt to minimize fetal dose, and prior to initiating treatment, fetal dose should be evaluated. The abdominal region should not be defined as part of the treatment volume^(1)^ and therefore, the fetus will only be exposed to out‐of‐field/peripheral dose. Peripheral dose (PD) is the absorbed dose received outside of the radiation treatment field. The principal components of PD are: 1) leakage radiation through the head of the treatment machine, 2) radiation scattered from the collimators and beam modifiers, and 3) radiation scattered within the patient from the irradiated volume (internal scatter).^(1)^

Some factors that affect the PD include distance from the radiation field edge,^5^, ^6^, ^7^ field size,^5^, ^6^, ^8^, ^9^, ^10^, ^11^, ^12^ and beam modifiers.^5^, ^9^, ^10^, ^11^ Depending on the location of the treatment site relative to the fetus, modifications to the radiation treatment plan can be applied to minimize the fetal dose. These changes may include reducing the size of the radiation apertures, changing the angle(s) of entry of the radiation beam(s), and selecting low‐energy X‐rays.^1^, ^2^ It has also been suggested that using flattening‐filter free (FFF) beams reduces PD by reducing leakage radiation to areas outside the treatment field.^13^, ^14^, ^15^, ^16^, ^17^, ^18^ However, even with these planning strategies, the fetus is still exposed to radiation from collimator scatter, gantry head leakage, and internal scatter.^1^, ^2^ To further reduce dose, the use of shielding material should be considered. Shielding material has been found to reduce fetal dose by upwards of 50%.^(2)^ Several studies suggested different designs for fetal shields^19^, ^20^, ^21^, ^22^, ^23^, ^24^, ^25^ and a summary of some designs have been provided in the report from AAPM TG 36.^(1)^ However, a number of these designs may be challenging to use with modern radiation therapy techniques such as volumetric arc therapy.

Prior to 2010, pregnant patients treated in the Department of Radiation Oncology at the University of Michigan were treated with a bridge‐style shield that would support eight 10.25 kg lead bricks. The bricks were manually positioned on the bridge while the patient was lying on the treatment couch, which raised safety concerns for both the patient and involved staff. The shield design also limited the treatment options to simple techniques. Due to these limitations and concerns, we designed and made a custom, mobile fetal shield.

The goal of this study was twofold. First, we designed and constructed a mobile fetal lead shield that would allow external beam treatments from multiple angles while minimizing the need to adjust the shield during the patient's treatment. Second, we created a comprehensive dataset of PD measurements with and without the fetal shield as a function of depth, field size, distance from the edge of the radiation field, collimator angle, and beam modifiers for common treatment protocols and modalities available in our department. The dataset allows users to assess PD and determine the effectiveness of the fetal shield for a variety of clinical sites and treatment techniques without requiring pretreatment phantom measurements.

## II. MATERIALS AND METHODS

**Figure 1:**
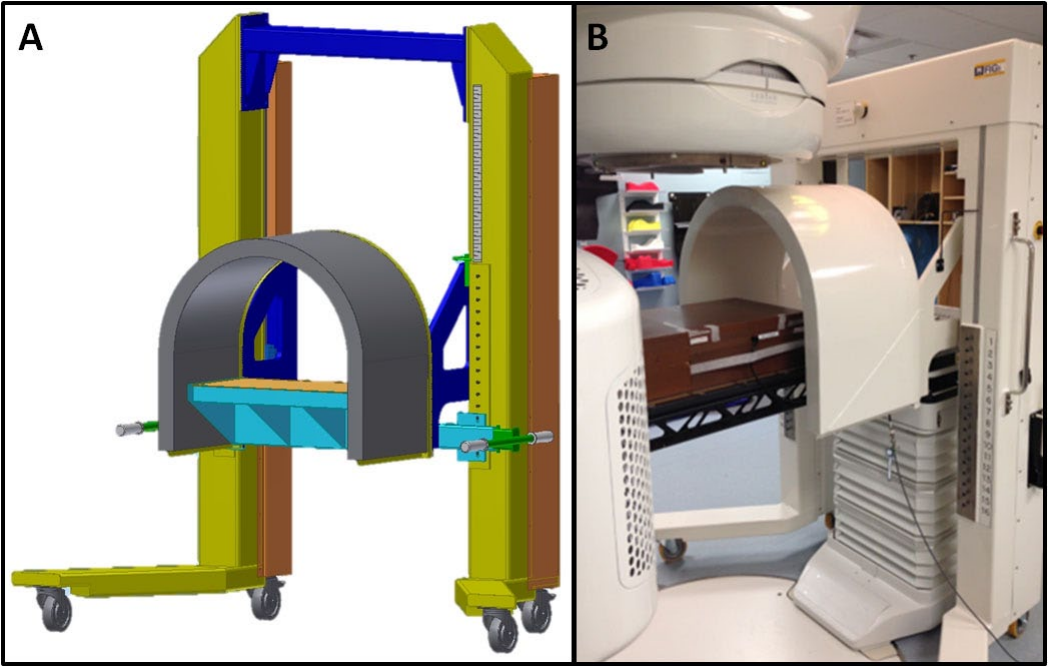
(a) A three dimensional schematic view of the fetal lead shield, and (b) a photo of the fetal lead shield positioned during the PD measurements.

A fetal shield was designed through a collaborative effort of three medical physicists in the Department of Radiation Oncology, a design and prototype consultant, and two biomedical engineers from the University of Michigan's Medical Innovation Center. The intention of the design was to provide shielding from multiple beam angles, so that more advanced treatment techniques could be used for pregnant patients. Another design aspect was to minimize adjustments of the shield during the patient's treatment. Consequentially, a 5‐cm thick U‐shaped lead shield with a detachable posterior shield was designed (the posterior shield was not used for phantom measurements and is not used clinically, due to collision‐related issues with the gantry). The shield was mounted on a mobile frame with motorized hydraulic jacks to raise and lower the shield (see Fig. 1). As suggested by AAPM TG 36,^(1)^ safety tests were performed on the shield and ancillary components at the manufacturing machine shop (Fame Industries Inc., Wixom, MI) and/or in our clinic. These tests included stability and tipping tests, emergency stops for the hydraulics, and checks of the safety pins that hold the shield once positioned.

To evaluate the effectiveness of the fetal shield in terms of dose reduction, point dose measurements were performed on a Varian Trilogy linear accelerator (Varian Medical Systems, Palo Alto, CA) equipped with a multileaf collimator (MLC). A stack of $40\times 110\times 20\;{\text{cm}}^{3}$ Solid Water (Solid Water model 457, Gammex/RMI, Milwaukee, WI) was used along with a Farmer‐type ionization chamber (Exradin A12, Standard Imaging, Middleton, WI) to estimate the range of doses that might be delivered to a fetus for a variety of situations. Radiation of varying square field sizes (6×6 to $30\times 30\;{\text{cm}}^{2}$) were delivered and point‐dose measurements were made at a range of depths (1.5 to 10.0 cm) and distances from the field edge (10 to 40 cm) (see Table 1). The distance from field edge was defined at the surface of the Solid Water phantom where the source to surface distance (SSD) was 100 cm. The PD measurements were normalized to the central axis dose (CAX) at a depth of 1.5 cm (the nominal depth of maximum dose for a 6 MV photon beam) for each respective field size. While the effects of depth and distance from the field edge were assessed for all field sizes, the effects of collimator angle (0° vs. 90°) and the tertiary MLC (retracted vs. shaped to the field aperture) were evaluated for only the $10\times 10\;{\text{cm}}^{2}$ field size. When rotating the collimator to 90°, the direction of motion of the lower jaws is parallel to the cranial‐caudal axis of the patient. When performing measurements at varying distances from the field edge, the position of the fetal shield was verified or adjusted such that it was adequately shielding the phantom and ion chamber, similar to the clinical setup.

**Table 1:** PD measurement conditions for the 6 MV photons.

			*Field Size (cm^2^)*			
	6×6	10×10	15×15	20×20	25×25	30×30
Depth (cm)	1.5, 5, 10	1.5, 5, 10	1.5, 5, 10	1.5, 5, 10	1.5, 5, 10	1.5, 5, 10
Distance from field edge (cm)	5, 10, 15, 20, 30, 40	5, 10, 15, 20, 30, 40	5, 10, 15, 20, 30, 40	5, 10, 15, 20, 30, 40	5, 10, 15, 20, 30, 40	5, 10, 15, 20, 30, 40
MLC	Shaped	Shaped, Retracted	Shaped	Shaped	Shaped	Shaped
Collimator Angle (degree)	90	0, 90	90	90	90	90

**Figure 2:**
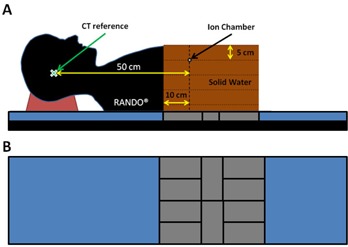
A sagittal schematic view (a) of the phantom used for the clinical treatment plan measurements. The phantom consisted of a RANDO phantom abutted to a $30\times 30\times 30\;{\text{cm}}^{3}$ Solid Water stack. An example ion chamber position is shown in the schematic. A schematic aerial view (b) of the posterior fetal lead shield. A series of 10 bricks with the thickness of 5 cm were aligned to cover an area of approximately $50\times 40\;{\text{cm}}^{2}$. The lead bricks were placed on the treatment couch at the approximate position of the fetus. The rest of the treatment couch was covered with 5 cm of Styrofoam to provide a flat surface for the patient/phantom.

Additionally, the height of the fetal shield was adjusted so that its highest point was within 10 cm of the phantom surface. To shield the fetus from posterior treatment fields, a series of ten 5 cm thick lead bricks were aligned on the treatment couch to cover an area of approximately $50\times 40\;{\text{cm}}^{2}$. The lead bricks were positioned so they would be directly under the phantom at approximately the level of the ion chamber. The rest of the treatment couch was covered with 5 cm of Styrofoam block to provide a flat surface for the phantom (see Fig. 2).

To compare the measured PD with the dose estimated in the treatment planning system (TPS), the Solid Water phantom was scanned (0.3 cm slice thickness) using a Philips 16 slice CT scanner (Royal Philips Electronics, Eindhoven, The Netherlands). The dataset was imported into the Eclipse TPS (version 11, Varian Medical Systems), and the PDs for the static fields listed in Table 1 were calculated as a function of depth and distance. Doses were calculated using a 0.25 cm grid size with the analytic anisotropic algorithm (AAA) version 11.0.31 and Acuros XB (AXB) algorithm version 11.0.30 in the TPS.

To estimate PD from clinically relevant treatment sites, a series of treatment plans were generated in the TPS. A RANDO anthropomorphic phantom (The Phantom Laboratory, Salem, NY) and a stack of $30\times 30\times 30\;{\text{cm}}^{3}$ Solid Water slabs, which was abutted inferiorly to the bottom of the RANDO phantom (see Fig.2(a)), were scanned with the CT and imported into the TPS. Treatment plans were generated for brain, head and neck, and lung treatment sites, using 3D conformal and intensity modulated radiation therapy (IMRT) techniques with 6 MV and 6 FFF beams (see Table 2). For the brain and lung treatment plans, contours were drawn and treatment plans were generated to provide suitable dose coverage to the planning target volume (PTV) while minimizing the dose to the surrounding organs at risk (OARs), based on the objectives and constraints specified in our standard clinical treatment planning directives. All plans were reviewed by an independent, qualified medical physicist. The head and neck plans were based on clinical plans generated for a pregnant patient who was treated in our department. A summary of the PTV and OAR dose limits used for planning each body site are listed in Table 3. The phantom was localized using kV on‐board imaging. PD measurements were then acquired for all treatment plans and techniques.

**Table 2:** Treatment plans that were generated to evaluate PD measurements.

*Body Site*	*TPS Technique*	*Energy*
Brain	3D	6 MV, 6 MV FFF
Brain	IMRT, VMAT	6 MV
Whole Brain	3D	6 MV
Head and Neck bilateral	3D, IMRT, VMAT	6 MV
Lung ‐ Left upper lobe	3D, IMRT	6 MV

TPS = treatment planning system.

**Table 3:** Dose limits and objectives for planning target volumes (PTVs) and organs at risk (OARs).

*Plan*	*Prescription Dose (Gy)*	*Number of Fractions*	*Structure*	*Dose Limits*
Brain	54	27	PTV	95% of IDL covers PTV
			Brainstem	Max to 0.1 cc less than 54 Gy
Whole Brain	30	10	PTV	95% of IDL covers PTV
			PTV	More than 100% of PTV volume receives at least 95% of prescription dose.
				No more than 0% of PTV volume receives more than 105% of prescription dose.
			Brainstem	Max to 0.1 cc less than 54 Gy
Head and Neck bilateral	66	33	Cord	Max to 0.1 cc less than 45 Gy
			Cord PRV 5mm	Max to 0.1 cc less than 50 Gy
			Esophagus	Mean dose less than 20 Gy
			Larynx	Mean dose less than 20 Gy
			Lips	V35.0 Gy> 5%
			PTV	Prescription dose cover 90% of PTV
Lung ‐ left upper lobe	60	30	Both Lungs minus GTV	V20.0 Gy> 35%
			Cord	Max to 0.1 cc less than 45 Gy

IDL = isodose line; PRV = planning organ at risk volume.

Initially, multiple measurements for each data point were acquired. Due to concerns with overheating and the length of time required to complete the more than 300 measurements, we examined the variation in our point measurements. The variation was less than 0.5%; single measurements for each data point were acquired for subsequent setups. To monitor the reproducibility of the measurements, repeat measurements for select data points were performed during the separate data collection sessions, which showed an average error of less than 3%.

## III. RESULTS

**Figure 3:**
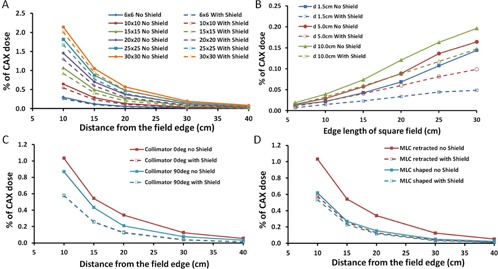
Measured PDs for static, square fields with a gantry angle of 0° normalized to the central axis dose at a depth of 1.5 cm. (a) PD measured at a depth of 10 cm as a function of distance from the field edge for different field sizes. These measurements were acquired with the MLC shaped to the field apertures and with a collimator rotation of 90°. (b) PD measured at a distance of 20 cm from the field edge at different depths as a function of field size with the MLC shaped around the field apertures and 90° collimator rotation. (c) PD measured with and without the fetal shield at a depth of 10 cm, plotted as a function of distance from the field edge with collimator angles of 0 and 90° for the field size of $10\times 10\;{\text{cm}}^{2}$. The field was shaped with the jaws, and the MLCs were fully retracted. (d) PD measured at a depth of 10 cm, plotted as a function of distance from the field edge for a $10\times 10\;{\text{cm}}^{2}$ field with the MLC shaped versus MLC retracted. Measurements were acquired at a collimator angle of 0°. For all plots, the solid lines represent the measured normalized PD in the absence of the fetal shield, while the dashed lines represent the measured normalized PD in the presence of the fetal shield.

A mobile custom fetal shield was designed to accommodate multi‐angle external beam radiation therapy. The measured PDs for square, static fields at a gantry angle of 0° are shown in Figs. 3(a) to 3(d). All PD measurements were normalized to the CAX dose at the depth of 1.5 cm, for the same field sizes. Figure 3(a) shows the PD at a depth of 10.0 cm as a function of distance from the field edge for varying field sizes. In the absence of the fetal shield, as the distance from the field edge increases from 10 to 40 cm, the PD decreases from 0.30% to 0.01% and 2.14% to 0.09% for field sizes of 6×6 and $30\times 30\;{\text{cm}}^{2}$, respectively. In the presence of the fetal shield, the PD for the $6\times 6\;{\text{cm}}^{2}$ field size decreases from 0.30% to 0.26% and 0.01% to 0.005% at distances of 10 and 40 cm from the field edge. For a $30\times 30\;{\text{cm}}^{2}$ field size, the PD decreases from 2.14% to 2.01% and 0.09% to 0.05% at distances of 10 and 40 cm from the field edge. Figure 3(b) shows the normalized PD measured at a distance of 30 cm from the field edge, with and without fetal shield, as a function of field size. As the depth increases from 1.5 to 10.0 cm, the PD increases from 0.01% to 0.02% and 0.14% to 0.20% for field sizes of 6×6 and $30\times 30\;{\text{cm}}^{2}$, respectively. With the fetal shield in place, a similar increase in PD was noted as the depth increases from 1.5 to 10 cm. For these depths and field sizes of 6×6 and $30\times 30\;{\text{cm}}^{2}$, the PD was observed to increase from 0.01% to 0.02% and 0.05% to 0.15%, respectively.

Figure 3(c) shows the effect of collimator rotation with and without the fetal shield for a $10\times 10\;{\text{cm}}^{2}$ field size with the MLCs retracted. In the absence of fetal shield, our results show that by rotating the collimator to 90° as compared to 0°, the PD decreases from 1.03% to 0.87% at a distance of 10 cm from the field edge. In the presence of the fetal shield, the PD is reduced from 1.03% to 0.57% at 10 cm with the collimator at 90°.

Figure 3(d) demonstrates the effect of the tertiary MLC on the PD with and without the fetal shield for a $10\times 10\;{\text{cm}}^{2}$ field size as a function of distance from the field edge. For this measurement, the collimator rotation was set to 0°. In the presence of the MLCs without the fetal shield, the PD decreased from 1.03% to 0.62% at a distance of 10 cm from the field edge. In the presence of both the MLCs and fetal shield, the PD was reduced from 1.03% to 0.57% at a distance of 10 cm from the field edge. A summary of the PD measurements relative to the CAX with and without the fetal shield are provided in 4, 5. These measurements can be used to estimate the fetal dose in the presence and absence of the fetal shield by determining the approximate distance from the fetus to the field edge, depth, the field size (using the most conservative field size for the treatment), and photon energy. For example, consider a pregnant patient who is prescribed a central axis dose of 50 Gy using 6 MV photons, and a maximum treatment field size of $20\times 20\;{\text{cm}}^{2}$. The dose to the fetus located at a distance of approximately 30 cm from the field edge can be estimated using 4, 5. Based on these tables, the estimated dose to the fetus with and without the shield at a depth of 10 cm can be calculated as 50 Gy×0.0012=0.06 Gy and 50 Gy×0.0009=0.04 Gy, respectively. Alternatively, if a patient is scheduled to be treated using one of the treatment techniques and sites listed in Table 6, one could estimate the dose to the fetus based on the PD estimates listed in Table 6. A summary of the dose estimates with and without the fetal shield to a fetal point approximated at a distance of 50 cm from the CT reference point illustrated in Fig. 2(a) for each of the measured, clinical plans and sites is shown in Table 7.

**Table 4:** Percent ratio of the measured PD normalized to the central axis (CAX) dose at depths of 1.5, 5, and 10 cm for varying field sizes and depths at distances ranging from 5 to 40 cm from the field edge in the absence of the fetal shield.

				*Distance From the Field Edge (cm)*					
*Field size (cm)*	*Depth (cm)*	*MLC Status*	*Collimator Angle (degree)*	*5*	*10*	*15*	*20*	*30*	*40*
6×6	1.5	Shaped	90	0.46	0.15	0.06	0.03	0.01	0.01
6×6	5	Shaped	90	0.65	0.20	0.08	0.04	0.02	0.01
6×6	10	Shaped	90	1.00	0.30	0.12	0.06	0.02	0.01
10×10	1.5	Retracted	0	2.22	0.87	0.55	0.31	0.12	0.06
10×10	5	Retracted	0	2.35	0.91	0.53	0.31	0.13	0.06
10×10	10	Retracted	0	2.86	1.03	0.54	0.34	0.12	0.05
10×10	1.5	Retracted	90	1.47	0.68	0.34	0.17	0.07	0.03
10×10	5	Retracted	90	1.75	0.73	0.37	0.18	0.07	0.03
10×10	10	Retracted	90	2.32	0.87	0.43	0.21	0.08	0.03
10×10	1.5	Shaped	0	0.94	0.31	0.15	0.10	0.04	0.04
10×10	5	Shaped	0	1.27	0.42	0.20	0.14	0.05	0.03
10×10	10	Shaped	0	1.91	0.62	0.27	0.20	0.05	0.03
10×10	1.5	Shaped	90	0.93	0.36	0.15	0.07	0.02	0.01
10×10	5	Shaped	90	1.26	0.46	0.21	0.10	0.03	0.01
10×10	10	Shaped	90	1.90	0.64	0.28	0.13	0.04	0.02
15×15	1.5	Shaped	90	1.47	0.59	0.30	0.15	0.04	0.02
15×15	5	Shaped	90	1.98	0.76	0.37	0.20	0.06	0.02
15×15	10	Shaped	90	2.96	1.07	0.50	0.26	0.07	0.03
20×20	1.5	Shaped	90	1.91	0.79	0.40	0.23	0.07	0.03
20×20	5	Shaped	90	2.63	1.04	0.51	0.29	0.09	0.03
20×20	10	Shaped	90	3.91	1.46	0.70	0.37	0.12	0.04
25×25	1.5	Shaped	90	2.24	0.95	0.48	0.28	0.11	0.04
25×25	5	Shaped	90	3.18	1.29	0.64	0.36	0.14	0.05
25×25	10	Shaped	90	4.74	1.82	0.88	0.47	0.16	0.06
30×30	1.5	Shaped	90	2.57	1.09	0.56	0.32	0.14	0.06
30×30	5	Shaped	90	3.70	1.50	0.75	0.42	0.16	0.07
30×30	10	Shaped	90	5.56	2.14	1.05	0.57	0.20	0.09

**Figure 4:**
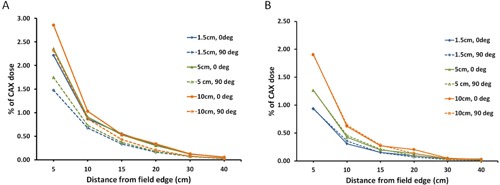
Measured PDs from a $10\times 10\;{\text{cm}}^{2}$ field normalized to the central axis dose (CAX) at a depth of dmax for a 6 MV photon beam with the MLC (a) retracted and (b) shaped to the field aperture. The PD for the 0° collimator rotation is shown in the solid lines, and the 90° rotation is shown in the dashed lines.

Figure 4 shows the effect of collimator rotation with and without field‐shaping with the MLC. To evaluate the effects of collimator rotation and the MLC, the measured PDs is normalized to the CAX at a depth of dmax. Figure 4(a) shows the PD with the MLC retracted, and Fig. 4(b) shows the PD with the MLC shaped around the field aperture at collimator rotations of 0° and 90°. The PD measurements were acquired for a $10\times 10\;{\text{cm}}^{2}$ field size at distances of 5 to 40 cm from the field edge.

**Figure 5:**
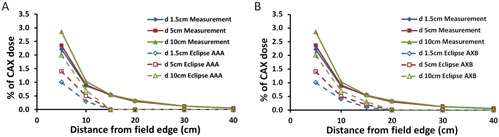
Measured and calculated PDs normalized to the central axis dose (CAX) at a depth of 1.5 cm. PD was measured at depths of 1.5, 5.0, and 10.0 cm as a function of distance from the field edge and compared to PD calculated with Eclipse's (a) anisotropic analytical algorithm (AAA) and (b) Acuros XB (AXB). All PD measurements and calculations were performed with a collimator angle of 0° and with the MLC retracted for the field size of $10\times 10\;{\text{cm}}^{2}$.

Figures 5(a) and 5(b) show the measured and calculated PDs normalized to the CAX at the same depth and field size as a function of distance from the field edge. The calculated PDs decrease as the distance from the field edge increases; however, compared to measurements for static fields, at 10 cm from the field edge the calculated PD was underestimated on average by 48% and 44% for the AAA and AXB algorithms, respectively. The underestimation of PD by the dose calculation algorithms increases as the distance from the field edge increases. At distances of ≥15 and 20 cm from the field edge for AAA and AXB, respectively, the calculated PD values were zero.

**Table 5:** Percent ratio of the measured PD normalized to the central axis (CAX) at depth of 1.5 cm dose for varying field sizes and depths at distances ranging from 5 to 40 cm from the field edge in the presence of the fetal shield.

				*Distance From the Field Edge (cm)*					
*Field size (cm)*	*Depth (cm)*	*MLC Status*	*Collimator Angle (degree)*	*5*	*10*	*15 20*	*15 20*	*30*	*40*
6×6	1.5	Shaped	90	0.32	0.12	0.06	0.03	0.01	0.00
6×6	5	Shaped	90	0.56	0.19	0.08	0.04	0.01	0.00
6×6	10	Shaped	90	0.88	0.26	0.11	0.05	0.01	0.00
10×10	1.5	Retracted	0	0.73	0.31	0.16	0.08	0.02	0.01
10×10	5	Retracted	0	1.21	0.43	0.21	0.10	0.03	0.01
10×10	10	Retracted	0	1.73	0.58	0.26	0.13	0.04	0.01
10×10	1.5	Retracted	90	0.71	0.30	0.15	0.07	0.02	0.01
10×10	5	Retracted	90	1.20	0.42	0.20	0.06	0.03	0.01
10×10	10	Retracted	90	1.73	0.57	0.25	0.12	0.04	0.01
10×10	1.5	Shaped	0	0.65	0.26	0.13	0.06	0.02	0.01
10×10	5	Shaped	0	1.10	0.39	0.18	0.09	0.02	0.01
10×10	10	Shaped	0	1.64	0.54	0.23	0.12	0.03	0.01
10×10	1.5	Shaped	90	0.65	0.26	0.13	0.06	0.01	0.01
10×10	5	Shaped	90	1.13	0.39	0.18	0.09	0.02	0.01
10×10	10	Shaped	90	1.66	0.54	0.23	0.11	0.03	0.01
15×15	1.5	Shaped	90	1.13	0.45	0.22	0.11	0.02	0.01
15×15	5	Shaped	90	1.81	0.66	0.31	0.16	0.04	0.02
15×15	10	Shaped	90	2.78	0.92	0.41	0.20	0.06	0.02
20×20	1.5	Shaped	90	1.67	0.62	0.32	0.17	0.03	0.02
20×20	5	Shaped	90	2.56	0.92	0.45	0.23	0.06	0.02
20×20	10	Shaped	90	3.76	1.29	0.60	0.30	0.09	0.03
25×25	1.5	Shaped	90	2.21	0.80	0.41	0.22	0.04	0.02
25×25	5	Shaped	90	3.33	1.20	0.59	0.31	0.08	0.03
25×25	10	Shaped	90	4.73	1.67	0.77	0.40	0.12	0.04
30×30	1.5	Shaped	90	2.60	0.96	0.50	0.27	0.05	0.02
30×30	5	Shaped	90	3.92	1.42	0.71	0.38	0.10	0.05
30×30	10	Shaped	90	5.45	2.01	0.94	0.48	0.15	0.05

**Table 6:** Percent ratio of the measured PD to the prescribed PTV dose for several clinically relevant body sites that were generated for this study. The PD data are shown both with and without the fetal shield.

	*PTV Volume (cm^3^)*	*Without Shield Ratio of PD to PTV Dose (%)*	*With Shield Ratio of PD to PTV Dose (%)*
Brain 3D	336	0.015	0.013
Brain 3D FFF	336	0.012	0.011
Brain IMRT	336	0.059	0.041
Brain VMAT	336	0.042	0.032
Whole Brain	2505	0.072	0.066
Head and Neck 3D	721	0.111	0.075
Head and Neck IMRT	721	0.278	0.258
Head and Neck VMAT	721	0.169	0.162
LUL 3D	211	0.117	0.085
LUL IMRT	211	0.138	0.084

LUL = left upper lobe of lung.

**Figure 6:**
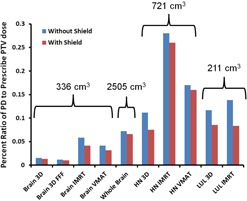
Percent ratio of the measured PD to the prescribed PTV dose for each body site. The red bars represent the percent ratios with the fetal shield in place, while the blue bars show the ratio in the absence of the shield. The volume of each PTV in cubic centimeters is noted above each treatment site. 3D = three‐dimensional conformal treatment planning; FFF = flattening filter free; IMRT = ntensity‐modulated radiation therapy; VMAT = volumetric‐modulated arc therapy; HN = head and neck; LUL = left upper lobe of lung.

Figure 6 shows the percent ratio of the measured PD to the prescribed planning target volume (PTV) dose for each body site. When considering brain radiotherapy, the presence of the fetal shield reduced this percent ratio, on average, from 0.04% to 0.03%. For head and neck and lung treatment plans the percent ratio of PD to the prescribed PTV dose was reduced, on average, from 0.19% to 0.17% and from 0.13% to 0.08%, respectively. A summary of the PDs measured for the clinically relevant plans are provided in Table 6. On average, the fetal shield reduced the percent ratio of the PD to prescribed PTV dose by 28% and 14% for three‐dimensional conformal and IMRT treatment plans, respectively, for all body sites considered in Table 2.

**Table 7:** The estimated fetal doses with and without the fetal shield based on the measured clinical plans listed in Table 2. A distance of 50 cm was maintained between the CT reference and the ion chamber used for these measurements, as shown in Fig. 2(a). The distance displayed in this Table is the measured distance between the center of the PTV and the ion chamber for each clinical setup.

	*Distance (cm)*	*Total PTV (or CAX) Dose (Gy)*	*PD Without Shield (cGy)*	*PD With Shield PD (cGy)*
Brain 3D	50	54	8.10	0.70
Brain 3D FFF	50	54	0.65	0.59
Brain IMRT	50	54	3.19	2.21
Brain VMAT	50	54	2.27	1.73
Whole Brain	50	30	2.16	1.98
Head and Neck 3D	40	66	7.33	4.95
Head and Neck IMRT	40	66	18.35	17.03
Head and Neck VMAT	40	66	11.15	10.69
LUL 3D	30	60	7.02	5.10
LUL IMRT	30	60	8.28	5.04

LUL = left upper lobe of lung.

## IV. DISCUSSION

Commissioning measurements were divided into PDs measured from simple, static square fields and clinically relevant treatment plans. For static fields, we found that in the absence of the fetal shield, the PD trends were consistent with measurements performed by other investigators.^5^, ^6^, ^7^, ^8^, ^9^, ^10^, ^11^, ^12^, ^26^ The PD decreased as the distance from the field edge increased due to the lower contribution of collimator scatter, head leakage, and patient scatter as determined by Stovall et al.^(1)^ Additionally, it was noted that at the same distance from the field edge, a higher PD was measured as the field size increased. This behavior results from an increase in head scatter (e.g., scatter from the collimators and flattening filter) and is consistent with previous findings.^1^, ^5^, ^6^, ^9^, ^10^, ^12^ As suggested in previous publications^5^, ^9^, ^10^ as well as the current study, when compared to measurements with a 0° collimator rotation, PD decreases when the collimator is rotated to 90°, where the direction of motion of the lower jaws are parallel to the cranial‐caudal axis of the patient. As such, the lower jaws provide additional shielding in the cranial‐caudal axis, which is relevant when considering treatment strategies to reduce fetal dose. Shaping the field aperture with the tertiary MLCs also reduced the PD by reducing the head leakage and head scatter.

Several studies recommend the use of MLCs combined with an appropriate collimator rotation.^9^, ^10^, ^20^, ^27^ Although a collimator rotation (90° vs. 0°) in the absence of MLC field‐shaping will reduce PD, our results showed that when the MLC shapes the field aperture, the application of a 90° collimator rotation is not clinically significant as was previously suggested by Mutic et al.^(5)^ This has important implications for treatment planning and supports the use of techniques such as field‐in‐field.

The measured treatment plans also demonstrated that as the distance between the treatment site and measurement point increased, the PD decreased. This was again due to the lower contribution of collimator scatter, head leakage, and internal scatter as the distance from the field edge increased. Additionally, as the volume of the PTV increased, the PD increased. This is due to the fact that larger PTVs require larger field apertures and as the field size increases, PD increases due to increased collimator scatter.^5^, ^6^, ^9^, ^10^, ^11^, ^12^, ^26^ When the plans for individual sites were compared, it was found that the measured PD for head and neck treatment plans were higher than the measured PD for lung treatment plans, even though the lung PTVs were closer to the PD measurement points. We believe this effect was due to the larger treatment volumes and higher beam modulation in the head and neck treatment plans that were considered for this study.

By introducing the fetal shield, the PD was found to decrease, as expected, and the PD reduction increased with increasing distance from the field edge. Additionally, the shielding effect was observed to be greater for larger field sizes (on average, 44% for a $30\times 30\;{\text{cm}}^{2}$ compared to 35% for a $6\times 6\;{\text{cm}}^{2}$ at a distance of 30 cm from the field edge). It was shown that in areas near the field edge, collimator and internal scatter are the main contributors to PD;^1^, ^12^ but at larger distances from the field edge (i.e., beyond 30 cm), head leakage is the dominant factor in PD. External shields are effective in reducing the contribution of head scatter and head leakage to PD. Therefore, at greater distances from the field edge and for larger field sizes, the fetal shield has a greater effect in PD reduction^1^, ^6^, ^12^

The largest potential sources of uncertainty in the PD measurements include the variation in individual point measurements and the variation in phantom and equipment setup, given the data collection was acquired over several months. As noted, multiple measurements for each data point were acquired early in the study, and the variation was determined to be less than 0.5%. To ensure the applicability of single measurements over the timeframe of the study, a subset of data points were acquired during each measurement session. The reproducibility of these repeat measurements at each session was consistent with the initial stable behavior of the linear accelerator.

The accuracy of PD calculations with the algorithms available in commercial TPSs are generally poor, as demonstrated by several investigators.^28^, ^29^, ^30^ Howell et al.^(30)^ observed that Eclipse's AAA version 8.6 underestimated PD on average by 40% over a distance of 3.75 to 11.25 cm from the field edge and up to 55% at a distance of 11.25 cm. This was confirmed in a previous study from our group,^(31)^ where we found that Eclipse's AAA version 11.0.31 and AXB version 11.0.30 underestimated PD on average, by 33% and 17%, respectively. Additionally, Huang et al.^(29)^ reported that the PD was underestimated by an average of 50% using Pinnacle version 9.0. The reported deviations between measured and calculated PDs is due in part to inadequate modeling of head leakage, head scatter and patient scatter.^(29)^ Kry et al.^(32)^ simulated a complete Varian Clinac 2100 linac head including all head shielding using MCNPX. They reported an average 16% dose difference between their Monte Carlo simulation and measurements for a 6 MV photon beam over a range of 50 cm from the field edge. Based on our current study, the observed difference between the measured and calculated PD increases as the distance from the field edge increases. Furthermore, the calculated PD approaches zero in the Eclipse TPS for distances beyond 12 cm from the field edge for the AAA and AXB algorithms. AAA underestimates dose due to the algorithm limiting the size of the dose matrix to minimize the number of calculation point used. AXB calculates dose to the entire volume but is limited by the input fluence margins. The reader can find a detailed summary on the limitations of the dose calculation in the Varian Eclipse Photon and Electron Algorithms Reference Guide.^(33)^ The newly designed fetal shield can reduce the PD for the pregnant patients and is safe and easy to set up. Due to its design, we are able to use a broad range of treatment techniques and beam angles without needing to shift the patient or shield during treatment. Additionally, the comprehensive PD dataset that was collected in this study allows our treatment team to assess fetal dose without the need to perform pre‐treatment PD measurements, which could potentially delay the patient's start date.

## V. CONCLUSIONS

We performed a comprehensive set of PD measurements for a range of static and clinically relevant treatment plans with and without the fetal shield. This dataset is intended to be used by the prescribing physician and treatment team to estimate fetal dose limits and help guide decisions on treatment technique as needed.

## ACKNOWLEDGMENTS

The authors would like to acknowledge the support of the University of Michigan's Fostering Innovation Grants (FIGs) for funding the design and build of the fetal shield. We would like to thank Michael Deininger and Laura McCormick from the University of Michigan Medical Innovation Center for our multiple discussions on the concept of the fetal shield, and the final design of the shield. We would like to thank Fame Industries Inc. (Wixom, MI) for constructing the frame and drive mechanism for the fetal shield. Lastly, we would like to acknowledge Dr. Kwok Lam for his many lively and fruitful discussions on shield designs and pertinent commissioning measurements.

## COPYRIGHT

This work is licensed under a Creative Commons Attribution 3.0 Unported License.
