# Revisiting fetal dose during radiation therapy: evaluating treatment techniques and a custom shield [JACMP, 17(5), 2016]

**DOI:** 10.1002/acm2.12191

**Published:** 2017-09-13

**Authors:** Amir M. Owrangi, Donald A. Roberts, Elizabeth L. Covington, James A. Hayman, Kathryn M. Masi, Choonik Lee, Jean M. Moran, Joann I. Prisciandaro

**Affiliations:** ^1^ Department of Radiation Oncology University of Michigan Ann Arbor MI USA

The authors would like to report an error in an entry for Table 7.^1^ Under the column labeled “PD without shield (cGy),” the first entry, 8.10, is off by a factor of ten. This entry should read 0.81.

**Table 7:** The estimated fetal doses with and without the fetal shield based on the measured clinical plans listed in Table 2. A distance of 50 cm was maintained between the CT reference and the ion chamber used for these measurements, as shown in Fig. 2(a). The distance displayed in this Table is the measured distance between the center of the PTV and the ion chamber for each clinical setup

	Distance (cm)	Total PTV (or CAX) dose (Gy)	PD without shield (cGy)	PD with shield (cGy)
Brain 3D	50	54	0.81	0.70
Brain 3D FFF	50	54	0.65	0.59
Brain IMRT	50	54	3.19	2.21
Brain VMAT	50	54	2.27	1.73
Whole brain	50	30	2.16	1.98
Head and neck 3D	40	66	7.33	4.95
Head and neck IMRT	40	66	18.35	17.03
Head and neck VMAT	40	66	11.15	10.69
LUL 3D	30	60	7.02	5.10
LUL IMRT	30	60	8.28	5.04

LUL, left upper lobe of lung.
